# Severe Hemorrhage from Cervical Cancer Managed with Foley Catheter Balloon Tamponade

**DOI:** 10.5811/westjem.2015.7.28058

**Published:** 2015-10-20

**Authors:** Tomohiro Sonoo, Ryota Inokuchi, Miyuki Yamamoto, Kensuke Nakamura, Susumu Nakajima, Naoki Yahagi

**Affiliations:** * The University of Tokyo Hospital, Emergency Medicine and Critical Care Medicine Department, Bunkyo-ku, Tokyo, Japan; † Hitachi General Hospital, Emergency Medicine Department, Hitachi-shi, Ibaraki, Japan

## CASE

A 67-year-old woman complaining of continuous fresh vaginal hemorrhage came to our emergency department in a pre-shock state. Examinations revealed an irregularly shaped mass in the uterus and active arterial bleeding. Emergent hysterectomy and interventional radiology were not immediately available. Foley catheter with 20mL water was inserted into the uterine cavity, then the balloon was pulled to obstruct the uterus output (Figure). Her vital signs became stabilized, and she was transferred to another hospital two days later.

## DIAGNOSIS AND DISCUSSION

A 67-year-old patient was diagnosed as Stage IIb cervical carcinoma. In this case, we controlled active bleeding from cervical cancer by balloon tamponade technique, which is frequently used in obstetric postpartum hemorrhage as a noninvasive and fertility-sparing procedure.^1^ Although devices specially made for obstetric hemorrhages are frequently used, less expensive and more easily accessible devices such as Foley catheters and condom catheters are also used.^2^,^3^ Only one case has ever been reported with this procedure used for hemorrhage from gynecologic malignancies.^4^ In this case, we were able to achieve sufficient tamponade effect using a single quite-small volume balloon, probably because the tumor itself and coagulated blood almost filled the uterine cavity.

Similar tamponade techniques are commonly used by emergency physicians for several emergency hemorrhages, such as vaginal, nasal, esophageal, or urethral hemorrhage, which usually require procedures performed by each specialty physician afterwards. Such balloon tamponade techniques are valuable as a bridge to specialty treatment because they can be conducted easily by emergency physicians using easily accessible devices.

## Figures and Tables

**Figure:**
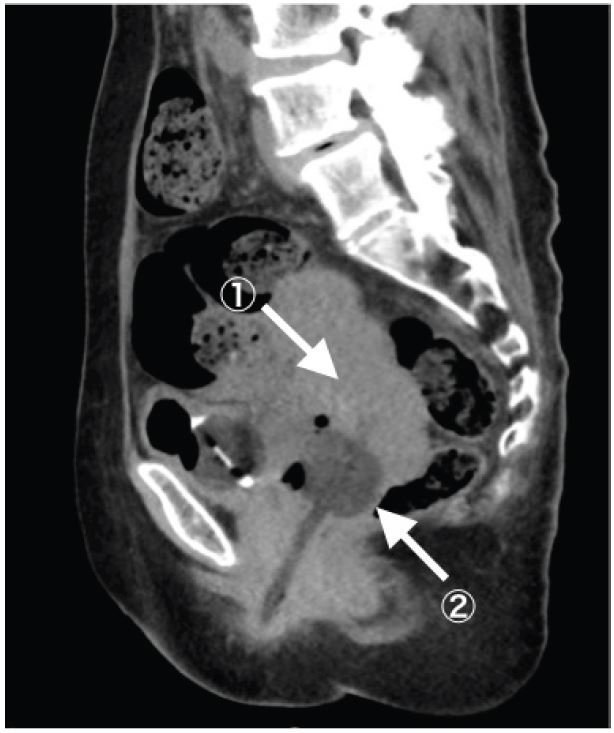
Successful balloon tamponade from the intrauterine mass using Foley catheter. Arrow 1: mass and coagulated blood filling the uterine cavity. Arrow 2: foley catheter balloon put at the uterine cervix.
